# Editorial: Technological solutions helping to train specialists' interviewing skills of possible victims and witnesses

**DOI:** 10.3389/fpsyg.2024.1406867

**Published:** 2024-05-02

**Authors:** Kristjan Kask, Gunn Astrid Baugerud, Renate Volbert

**Affiliations:** ^1^School of Natural Sciences and Health, Tallinn University, Tallinn, Estonia; ^2^Department of Social Work, Child Welfare and Social Policy, Faculty of Social Science, Oslo Metropolitan University, Oslo, Norway; ^3^Psychologische Hochschule Berlin, Berlin, Germany

**Keywords:** investigative interviewing, serious gaming, avatar, adult education, adult learning

Newly adopted The Mendez Principles (Association for the Prevention of Torture, 2022) form a framework how investigative interviews should be conducted worldwide. Just recently a white paper with recommendations based on the research regarding forensic child interviewing was published by the European Association of Psychology and Law (Korkman et al., 2023). The white paper clearly points out that interviews in cases of suspected child abuse should be conducted according to evidence-based interview protocols such as the NICHD interview protocol (Orbach et al., 2000). In addition, it is recommended that “the interviewers should receive specialized training, including continuous assessment and feedback on their interviewing style for quality assurance” (Korkman et al., 2023).

Pompedda (2018) points out that short and intensive theoretical trainings are (probably) the most common training formats. When practice sessions are included then in these trainings actors are used to portray the witnesses the trainees have to interview to practice newly learned skills in a format of a role play. However, it is known that the effect of such trainings will fade quickly if the feedback to the interviewing skills is not continuous (Lamb et al., 2002). Thus, using a structured interview protocol such as the NICHD protocol accompanied with extensive feedback increase the quality of the interviews, however, the investigators' performance declines when the feedback will be not given anymore (Lamb et al., 2002). Lamb (2016) points out that a knowledge transfer problem exists where interviewers are aware of the best-practice techniques but still fail to apply them in their interviews. One crucial point in here would be obtaining feedback on the quality of the interviews to consolidate deep learning processes.

During the last decade or so, several technological solutions have been created to practice interviewing skills. Powell et al. (2016) were among the first that started to use technological solutions and an eLearning curriculum for enhancing child interview training. They initiated the utilization of a mechanized child avatar in 2009, that allows trainees to interact by selecting from a set of tailored, predefined questions, to which the avatar respond (Guadagno and Powell, 2012). Subsequently, the efficacy and the value of such online training extends beyond conventional classroom instruction and is well-supported by a body of research, including studies by Benson and Powell (2015), Powell et al. (2016), and Brubacher et al. (2022). Following this development, additional avatar-based solutions have emerged (Pompedda et al., 2015; Haginoya et al., 2021), along with Artificial Intelligence (AI)-powered avatars (Baugerud et al., 2021; Salehi et al., 2022).

The present Research Topic includes four experimental articles regarding investigative interviews of children. Each of these papers contributed to the literature by expanding our understanding of how technological solutions could help us increase the quality of these interviews.

Segal et al. were interested in associations between emotions and psychophysiological states when formulating questions in simulated interviews of child avatars. Sixty psychology students in Lithuania conducted two interviews with child avatars while their emotions, galvanic skin response (GSR) resistance and heart rate (HR) were registered. They found that closed questions (in comparison with open questions) were preceded by more facially observable anger, higher GSR resistance and lower HR. These results indicate that the constructs under observation can drive confirmation bias in formulating questions. Thus, the interviewers who have difficulties regulating themselves emotionally may interview children in a more biased way. Feedback also to interviewers' emotionality and expressed emotions could be a solution to solve this issue.

Kask et al. examined whether knowledge transfer effect will be present among police investigators. Twenty-two Estonian investigators participated the experiment. Half of the investigators received feedback during four simulated child avatar interviews. The rest of the investigators received first no feedback during four avatar interviews and then received feedback for the subsequent four interviews. Investigators gave the researchers access to actual police interview transcripts with actual child victims and witnesses of physical and sexual abuse. Receiving feedback increased the proportion of recommended questions (i.e., invitations, open questions) in avatar training and also in the actual police interviews (29% increase in asking recommended questions) after the avatar training with feedback. The results suggests that this solution can be used as a tool in investigative interviewing to train police officers.

Haginoya et al. investigated the agreement of question coding between human operators and automated classification using AI (artificial intelligence). They created an AI model (AI Avatar) that classified the interviewer's questions and selected appropriate answers without human assistance. Forty-two Japanese professionals conducted two simulated child avatar interviews and were provided wither no intervention, feedback, or modeling after the first interview. The percentage of agreement in coding question types (recommended vs. non-recommended) was 72%. The results indicate higher-than-chance-level classification accuracy showing the potential for scalable training in the future to increase the proportion of recommended question types in interviewers' questions.

Røed et al. continued to study AI technology more in depth. There were interested in applying AI technology in the child witness investigative interviewing context using a ChatBot child avatar trained on well-designed mock investigative interviews Thirty Norwegian students were divided into two groups, one received automated feedback from the AI-system and the other did not. AI identified the question types. The results indicated high agreement among the raters in coding open-ended, cued recall and closed questions. Furthermore, the level of agreement between the AI-based classification model and human coders were high showing an excellent agreement. Those participants who received feedback showed a higher improvement in open-ended questioning than those who did not receive feedback. It can be concluded that their experiment provides preliminary support for the use of self-run child avatar ChatBots. When integrated with the feedback function, this type of training would be beneficial in increasing the quality of the interviewing skills.

In conclusion, this Research Topic extends our understanding of how technology can help to training investigative interviewing skills of the investigators regarding child witnesses. Although the papers focused on child witnesses, there is also a room to improve in a similar way of adult victims and witnesses' interviews by the police officers as suggested by Tohvelmann and Kask (2022).

## Author contributions

KK: Writing – original draft. GB: Writing – review & editing. RV: Writing – review & editing.
